# History of the Inoculation of the Cow-Pox

**Published:** 1799-06

**Authors:** Edward Jenner


### Medical and Physical Journal.

VOL. I.] JUNE, 1799. [NO. IV.

### *History of the Inoculation of the Cow-pox.*

[Continued from page 217 of our last.]

*Further Observations on the Variolæ Vaccinæ, or Cow-pox: By* EDWARD JENNER, M. D. F. R. S. F. L. S, *&c.* 4*to.* 64 *Pages.* 2*s.* 6*d.* Law, *&c.*

IN his first publication, Dr. JENNER's principal object appears to have been to excite an extensive inquiry into the origin, nature, and effects of the cow-pox. In this he has completely succeeded.

There is not, perhaps, in the annals of medicine, to be found an example of an experiment or enquiry, where the life and health of such numbers already born, and of all to be born, were implicated, that has been taken up more generally, received more candidly, or conducted more prudently, than this concerning the cow-pox.

In this second work it appears to be the author's principal concern, to guard the inquiry against misrepresentations, and inferences drawn from insufficient premises.

The following are the most important subjects introduced in it, viz.

1. The means of distinguishing the *real cow-pox* in the brute or human subject; and thereby of preventing the "unpleasant consequences" that might ensue, from confounding it with a *spurious disease*, which may have no power to defend the patients from the contagion of the small-pox.

2. As the inoculated cow-pox very rarely produces any anxiety in the patient or practitioner, unless perhaps in a few instances, respecting the local inflammation of the arm; Dr. Jenner solicits the attention of his brethren to the best mode of treating this species of erythema; and suggests some hints that the constitutional disease, on this, and other occasions, may be much modified by a modification of the local affection.

3. Some additional arguments are adduced to prove that the variolæ vaccinæ are derived from the horse; and that the disease is contagious only by inoculation.

4. An inquiry into the caufe of the variety which has been obferved in a few cafes of children inoculated in London, where an eruption appeared on the body, &c. refembling the fmall-pox.

Dr. Jenner introduces his work with the following account of his views in its publication.

"Although it has not been in my power to extend the inquiry into the caufes and effects of the variolæ vaccinæ much beyond its original limits, yet, perceiving that it is beginning to excite a general fpirit of inveftigation, I think it of importance, without delay, to communicate fuch facts as have fince occurred, and to point out the fallacious fources from whence a difeafe, imitative of the true variolæ vaccinæ, might arife, with the view of preventing thofe who may inoculate, from producing a fpurious difeafe; and further, to enforce the precaution fuggefted in the former treatife, on the fubject of fubduing the inoculated puftule as foon as it has fufficiently produced its influence on the conftitution. From a want of due difcrimination of the real exiftence of the difeafe, either in the brute or in the human fubject, and alfo of that ftage of it in which it is capable of producing the change in the animal economy, which renders it unfufceptible of the contagion of the fmall-pox, unpleafant confequences might enfue, the fource of which, perhaps, might not be fufpected by one inexperienced in conducting fuch experiments.

"Ere I proceed, let me be permitted to obferve, that truth, in this and every other phyfiological inquiry that has occupied my attention, has ever been the object of my purfuit; and fhould it appear in the prefent inftance, that I have been led into error, fond as I may appear of the offspring of my labours, I had rather fee it perifh at once, than exift and do a public injury.

"I fhall proceed to enumerate the fources, or what appear to me as fuch, of a fpurious cow-pox.

"1ft. That arifing from puftules on the nipples or udder of the cow, which puftules contain no fpecific virus.

"2dly. From matter (although originally poffeffing the fpecific virus) which has fuffered a decompofition, either from putrefaction, or from any other caufe lefs obvious to the fenfes.

"3dly. From matter taken from an ulcer in an advanced ftage, which ulcer arofe from a true cow-pox.

"4thly. From matter produced on the human fkin from contact with fome peculiar morbid matter generated by a horfe.

"On

"On theſe ſubjects I ſhall offer ſome comments.

"One of the firſt objects of this purſuit ſhould be, to learn how to diſtinguiſh with accuracy between that peculiar puſtule which is the *true* cow-pox, and that which is ſpurious. Until experience has determined this, we view our object through a miſt. Let us for inſtance ſuppoſe, that the ſmall-pox and the chicken-pox were at the ſame time to ſpread among the inhabitants of a country, which had never been viſited by *either of theſe diſtempers*, and where they were quite unknown before; what confuſion would ariſe! The patient who had gone through the chicken-pox to any extent, would feel equally eaſy with regard to his future ſecurity from the ſmall-pox, as the perſon who had actually paſſed through that diſeaſe. Time and future obſervation would draw the line of diſtinction. So I preſume it will be with the cow-pox, until it is more generally underſtood. All cavilling therefore, on the mere report of thoſe who *tell us* they have had this diſtemper, and are afterwards found to be ſuſceptible of the ſmall-pox, ſhould be ſuſpended. To illuſtrate this, I beg leave to give the following hiſtory:"

Dr. Jenner then details a caſe of a young woman, who took the cow-pox, as ſhe ſuppoſed, by milking infected cows, and afterwards experienced the ſmall-pox. This caſe is ſo ſimilar to thoſe which have been lately mentioned in converſation, as proofs of the inefficacy of the variolæ vaccinæ to protect the ſyſtem from the ſmall-pox, that little or no doubt can remain of their identity; and ſufficiently proves, on what ſlight evidence popular rumours are commonly founded. Dr. Jenner obſerves:—"Had any one converſant with the habits of the diſeaſe, heard this hiſtory, he would have had no heſitation in pronouncing it a caſe of *ſpurious* cow-pox.

"This is, perhaps, the moſt deceptious form in which an eruptive diſeaſe can be communicated from the cow, and it certainly requires ſome attention in diſcriminating it. The moſt perfect criterion by which the judgment may be guided is, perhaps, that adopted by thoſe who attend infected cattle. Theſe white bliſters on the nipples, ſay they, *never eat into the fleſhy parts*, like thoſe which are commonly of a blueiſh caſt, and which conſtitute the *true cow-pox*, but that they affect the ſkin only, quickly end in ſcabs, and are not nearly ſo infectious." P. 8.

On the *ſecond* ſource of diſappointments and falſe inferences, Dr. Jenner ſays:—"I conſider it of very great importance, and I could wiſh it to be ſtrongly impreſſed on the minds of all who may be diſpoſed to conclude haſtily on my obſervations, that *imperfect variolous matter* will, by inoculation, produce a diſeaſe, but not ſuch an one as will render the ſyſtem unſuſceptible of of the *ſmall-pox*." This opinion is ſupported by a reference to a conſiderable number of facts, ſome mentioned in the author's firſt treatiſe, others in the Memoirs of the Medical Society of London, vol. iv. and ſeveral in the preſent publication.

*Thirdly.* "That the firſt-formed virus, or what conſtitutes the true cow-pox puſtule, invariably poſſeſſes the power I have aſcribed to it, namely, that of affecting the conſtitution with a ſpecific diſeaſe, is a truth that no ſubſequent occurrence has yet led me to doubt. But as I am now endeavouring to guard the public as much as poſſible againſt erroneous concluſions, I ſhall obſerve, that when the puſtule has degenerated into an ulcer (to which ſtate it is often diſpoſed to paſs, unleſs timely checked), I ſuſpect that matter poſſeſſing very different properties, may ſooner or later be produced; and although it may have paſſed that ſtage, wherein the ſpecific properties of the matter ſecreted are no longer preſent in it, yet, when applied to a ſore, as in the caſual way, it might diſpoſe that ſore to ulcerate, and from its irritation the ſyſtem would probably become affected; and thus, by aſſuming ſome of its ſtrongeſt characters, it would imitate the genuine cow-pox." P. 20.

*Fourthly.* "Whether the cow-pox is a ſpontaneous diſeaſe in the cow, or is to be attributed to matter conveyed to the animal, as I have conceived, from the horſe, is a queſtion, which though I ſhall not now attempt fully to diſcuſs, yet I ſhall digreſs ſo far as to adduce ſome further obſervations, and to give my reaſons more at large, for taking up an opinion that to ſome has appeared fanciful. The aggregate of theſe obſervations, though not amounting to poſitive proof, forms preſumptive evidence of ſo forcible a kind, that I imagine it might on any other perſon have made the ſame impreſſion it did on me, without fixing the imputation of credulity." P. 21.

Dr. Jenner then introduces a number of conſiderations, the weight of which had induced him to adopt this opinion, and which we think abundantly ſufficient to juſtify his giving it to the public.

The next ſubject, which Dr. Jenner thinks of ſufficient importance to demand the particular attention of medical men, is the effect of local applications to the inciſed part. P. 31—59.

"Conceiving theſe caſes to be important, I have given them in detail; firſt, to urge the precaution of uſing ſuch means as may ſtop the progreſs of the puſtule; and, ſecondly, to point out (what appears to be the fact) that the moſt material indiſpoſition, or at leaſt that which is felt moſt ſenſibly, *does not ariſe primarily from the* FIRST ACTION *of the virus on the conſtitution; but that it often comes on, if the puſtule is left to chance, as a ſecondary diſeaſe.*

Although

"Although the application (ung. hydr. nitr.) I have mentioned in the cafe of Mary Hearn (p. 31), proved fufficient to check the progrefs of ulceration, and prevent any fecondary fymptoms, yet, after the puftule has duly exerted its influence, I fhould prefer the deftroying it quickly and effectually to any other mode. The term *cauftic*, to a tender ear (and I conceive none will feel more interefted in this inquiry than the anxious guardians of a nurfery), may found harfh and unpleafing; but every folicitude that may arife on this account will no longer exift, when it is underftood, that the puftule, in a ftate fit to be acted upon, is then quite fuperficial, and that it does not occupy the fpace of a filver penny. I mention efcharotics for ftopping the progrefs of the puftule, becaufe I am acquainted with their efficacy; probably more fimple means might anfwer the purpofe quite as well—fuch as might be found among the mineral and vegetable aftringents." We are authorized to fay, that Goulard's extract has been found to fucceed completely.

Dr. Jenner prefents his readers with feveral facts, tending to prove, that the arrefting the variolous or vaccine difeafe in its progrefs, as foon as the fyftem is perceived to fympathife with the local affection, does not diminifh the future fecurity of the patient.

He inculcates the idea (fuggefted in his 'Inquiry,' &c. p. 54), that the difeafe may be much modified by attention to the local affection; and hints an opinion, that feveral *varieties*, of both difeafes, may be produced by a fkilful attention to the incifed part of the arm (p. 37); and at p. 39, the author fums up this part of the fubject with the following obfervations:— "Seeing that we poffefs the means of rendering the action of the fores mild, which, when left to chance, are capable of producing violent effects— and feeing, too, that thefe fores bear a refemblance to the fmall-pox, *efpecially the confluent*, fhould it not encourage the hope, that fome topical application might be ufed with advantage, to counteract the fatal tendency of *that difeafe*, when it appears in this terrific form? At what ftage, or ftages of the difeafe, this may be done with the moft promifing expectation of fuccefs, I will not pretend now to determine; I only throw out this idea as the bafis of future reafoning and experiment."

Dr. Jenner next fuggefts fome improvements in the *manner* of inferting the virus in inoculation, and in diftinguifhing the beft time for taking the matter from the puftule, and adds:—"In the prefent early ftage of the inquiry (for early it certainly muft be deemed), before we know for an abfolute certainty, how foon the virus of the cow-pox may fuffer a change in its fpecific properties, after it has quitted the limpid ftate it poffeffes when forming a puftule, it would be prudent for thofe who have been inoculated with it, to fubmit to variolous inoculation." P. 42.

There

There obviously appears to be a difference between several diseases in London, and the same diseases in the country; as erysipelas, scrofula in children, intermittents, &c. and there has appeared a difference in the cow-pox. Dr. Jenner says:—"The principal variation perceptible to me in the action of the vaccine virus generated in London, from that produced in the country, was its proving more certainly infectious, and giving a less disposition in the arm to inflame. There appears also a greater elevation of the pustule above the surrounding skin. In my former cases, the pustule produced by the insertion of the virus was more like one of those which are so thickly spread over the body in a bad kind of confluent small-pox. This was more like a pustule of a distinct small-pox, except that I saw no instance of pus being formed in it, the matter remaining limpid till the period of scabbing." P. 62.

Another mark of distinction between the variolous and vaccine disease is mentioned, with a case, in p. 63; viz. that the measles, which have been observed to suspend the action of variolous matter, do not suspend the progress of the cow-pox. The author concludes his pamphlet with the following paragraph, the assertions of which, we are well assured, are corroborated by the experience of every person who has practised the vaccine inoculation:—

"The very general investigation that is now taking place, chiefly through inoculation (and I again repeat my earnest hope, that it may be conducted with that calmness and moderation which should ever accompany a philosophical research), must soon place the vaccine disease in its just point of view. The result of all my trials with the virus on the human subject, has been uniform. In every instance, the patient who has felt its influence, has completely lost the susceptibility for the variolous contagion; and these instances are now become numerous: I conceive that, joined to the observations in the former part of this paper, they sufficiently preclude me from the necessity of entering into controversies with those who have circulated reports adverse to my assertions, on no other evidence than what has been casually collected."

